# For Better or Worse: Self-reported Changes in Kratom and Other Substance Use as a Result of the COVID-19 Pandemic

**DOI:** 10.1177/11782218221123977

**Published:** 2022-09-28

**Authors:** Jeffrey M Rogers, Kirsten E Smith, Destiny Schriefer, David H Epstein

**Affiliations:** 1 National Institute on Drug Abuse Intramural Research Program, Baltimore, MD, USA; 2 San Diego State University/University of California San Diego Joint Doctoral Program in Clinical Psychology, San Diego, CA, USA

**Keywords:** Kratom, *Mitragyna speciosa*, COVID-19, opioids, cannabis

## Abstract

**Background:**

Kratom is taken to self-treat pain and symptoms of psychiatric disorders, including substance-use disorders (SUDs) and opioid withdrawal. Before COVID-19, kratom use was increasing in the US, however, there are few published data on whether that trend continued during the COVID-19 pandemic, which could have affected kratom use in multiple ways.

**Aim:**

To examine COVID-19-related changes in kratom use and how these changes were experienced, relative to changes in other commonly used substances.

**Methods:**

Using Amazon Mechanical Turk, 2615 evaluable surveys were completed between September 2020 and March 2021. Responses from past-month and past-year kratom-using adults (N = 174) indicating changes for the better or worse were examined using generalized linear mixed effects models, and relevant open-text responses (n = 85) were thematically coded.

**Results:**

For kratom 33% (n = 58) reported a Covid-related increase and 24% (n = 42) reported a Covid-related decrease. Controlling for changes in amount used, alcohol (OR = 5.02), tobacco (OR = 4.72), and nonmedical opioid use (OR = 3.42) were all more likely to have changed for the worse, compared with kratom use. Relative to decreases in kratom use, decreases in alcohol (OR = 3.21) and tobacco (OR = 6.18) use were more likely to be changes for the better. Cannabis use was the only substance to display a probability lower than 50% of being a decrease for the better, and of the increases, cannabis use displayed the highest probability of being for the better.

**Conclusions:**

Increases in kratom and cannabis use were less likely than alcohol and tobacco to be reported as changes for the worse, and decreases in kratom and cannabis use were more likely than alcohol and tobacco to be reported as changes for the better. These findings indicate that people differently conceptualize their relationships with kratom and cannabis, compared to their relationships with alcohol and tobacco.

## Introduction

### The COVID-19 pandemic and drug use in the US

COVID-19—the disease itself and the physical-distancing measures needed to limit its spread—disrupted employment, education, commerce, recreation, and healthcare access; all of which contributed to psychological distress.^1-8^ One downstream effect, corroborated by some survey evidence, has been changes in demand for and consumption of psychoactive drugs.^2,9-13^ Available evidence indicates that the proportion of people reporting cannabis, opioid, and stimulant use increased as a result of the pandemic, and though evidence is mixed regarding the prevalence of alcohol use, reports of solitary drinking, binge drinking, and drinking to cope with psychosocial stress did increase as a result of the pandemic.^1^

The coincidence of COVID-19 with the ongoing US opioid epidemic, 2 crises in public heath, may have created unique conditions for exacerbation of each crisis by the other.^14,15^ Opioid misuse and overdoses increased in some regions correspondingly with decreased mobility and social interaction during COVID-19, even among people receiving medication for opioid-use disorder (MOUD).^16,17^ Opioid misuse following the pandemic’s onset is one part of a larger complex of problematic pandemic-related dynamics, including psychological distress, psychiatric disorders, and decreases in protective factors or coping capacity.^2,18-21^ Particularly concerning are reports of decreased access to or disruptions of traditional forms of treatment or support for SUDs and other psychiatric problems.^22-26^ Even with a compensatory uptick in virtual support services, people’s perceived self-confidence to maintain treatment goals may have been disrupted by loss of access to conventional in-person support.^27,28^

Disruptions also extended to the realm of how drugs themselves were marketed and accessed, with shifts to greater online purchasing and increased use of some unregulated substances.^29-31^ This may have been particularly true during the early months of the pandemic.^32^

### Kratom’s place amidst the US opioid crisis

Even prior to COVID-19, the US opioid crisis narrative had taken a subtle twist: there was increased availability and use of kratom (*Mitragyna speciosa*), a plant with psychoactive properties, especially among people with chronic pain, active or remitted SUDs, or iatrogenic physical dependence on opioids.^33-39^ Kratom’s complex alkaloid profile and pharmacology remain far from understood, but 2 of its main constituents, mitragynine and 7-hydroxymitragynine, act as partial, seemingly “biased” agonists at μ-opioid receptors and are believed to be involved in kratom’s analgesic effects.^40-44^ (We use the term “biased” with quotation marks in light of findings that “biased” opioid actions may not work by the mechanism originally proposed.^45,46^ ) These are only 2 of over 40 bioactive alkaloids in kratom, many of which also have non-opioid mechanisms of action that may contribute to not only analgesia, but anxiolytic, antidepressant, and possibly antipsychotic effects, making it premature and probably incorrect to classify or conceptualize kratom as *only* an opioid.^43,47-50^ Nonetheless, for people with OUD or iatrogenic physical dependence on opioids, kratom has been used successfully to self-treat opioid withdrawal and serve as a short- and long-term opioid substitute.^35,36,38,51-53^

Kratom was already being used by a large number of Americans when COVID-19 led to sweeping public-health mandates and shutdowns in the US; estimates of that number range from under 1 million to over 15 million.^54-56^ Kratom is legal in 44 US states and can be purchased in retail stores and online.^32,57^ During the early months of the pandemic, some kratom vendors reportedly increased production to counter possible disruptions and to meet demand by consumers who may have wanted to stock up.^32^ Given that many people who use kratom are doing so to self-treat medical or psychiatric symptoms,^35,36,51,58,59^ disruptions in the US kratom market could lead to significant consequences for regular kratom consumers.

On the other hand, recent evidence suggests that kratom is increasingly being used recreationally and as a performance enhancing “nootropic” substance.^3,4^ Coupled with this trend, there is also evidence that kratom’s increasing prevalence and popularity is growing the fastest among young, non-Hispanic White, middle-class men.^5^ As the demography and motivations behind kratom use change, reported kratom use patterns and kratom-related consequences may also change.

It is not yet clear how people modified their kratom use in response to kratom availability, increased psychosocial stress, or social isolation. Examining people’s reasons for changed drug usage and their perceptions of the consequences may better elucidate the relationships that people have with kratom, compared to other commonly used substances. Additionally, the question is of more than historical interest; pandemic-related shutdowns in the US are likely to recur, and, although future shutdowns will not be identical to the shutdown of 2020, there are lessons to be learned.

We aimed to examine whether people reported changes in kratom use similar to or different from other substances (ie, alcohol, tobacco, cannabis, nonmedical stimulants, and nonmedical opioids), we examined data from US adult respondents who reported past-year and past-month kratom use as part of their participation in a larger online survey. Our goals in the analyses reported here were to: (1) characterize this subsample of kratom-using adults, (2) determine self-reported changes in amounts of kratom use due to COVID-19, relative to other most commonly used substances, and (3) using closed- and open-ended questions, to examine perceptions of use patterns (with or without dosage changes) as having changed “for the better” or “for the worse.” To contextualize self-report, we examined changes in kratom use relative to the use of other substances (ie, alcohol, tobacco, cannabis, nonmedical opioids, and nonmedical stimulants) used most often by our respondents.

## Methods

### Participant screening and recruitment

For the larger study from which this sample was drawn, we recruited and screened people for study inclusion using the online crowdsourcing platform Amazon Mechanical Turk (MTurk). MTurk is increasingly used for obtaining national convenience samples in behavioral research; we used several of the mechanisms it offers for increasing data quality and validity.^60-67^

People were eligible for participation if they were: ⩾18 years or older, US residents, English language proficient, and had ⩾100 completed MTurk human intelligence tasks (HITs), indicating greater reliability and MTurk experience.^63^ They also had to endorse past 6-month drug use (⩾1 day of use during the 6 months prior to screening) for one of the following 2 categories: (1) *alcohol only* (nicotine and caffeine use permitted, but no other past 6-month drug use permitted); (2) *opioids or psychostimulants* (the data in this paper are from the second group, as explained below). Our sampling strategy was intended to capture one group whose drug use might be seen as more socially normative and another group whose drug use might be seen as more deviant or stigmatized (though we expected heterogeneity in each group). People qualified for the opioid/psychostimulant group if they used licit opioids (prescription opioid analgesics, prescribed methadone, and/or prescribed buprenorphine), illicit opioids (heroin, fentanyl, nonmedical/diverted prescription opioids, and/or nonmedical/diverted methadone or buprenorphine), kratom (which we considered an opioid with variable legality), or illicit psychostimulants (powder or crack cocaine, synthetic cathinones, “street” methamphetamine, 3,4-methylenedioxymethamphetamine (MDMA), or diverted prescription psychostimulant medications).

### Data collection

**Figure 1.**
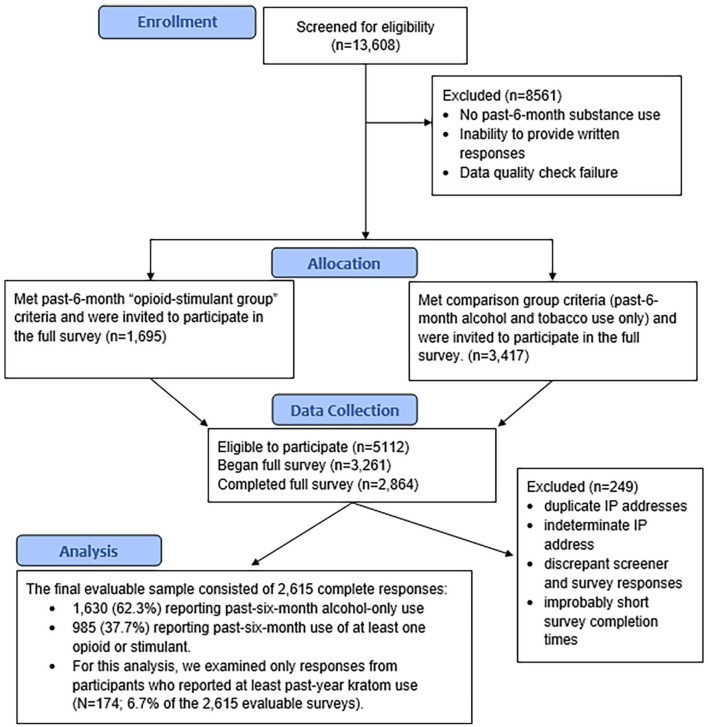
Participant screening, data collection, and data analysis flow chart.

A visual description of participant recruitment/screening, data collection, and data analysis is provided in Figure 1. MTurk workers completed 13 608 screening surveys. Of those who completed screening, 3417 (25.1%) met inclusion for the past-6-month “alcohol group” and 1695 (12.5%) met inclusion for the past-6-month “opioid-stimulant group” (which included kratom). Eligible workers who completed the full survey on Qualtrics were compensated $7.25. To help ensure validity, quality checks were programmed into the survey. Failing 3 quality checks, or exceeding the 4-hour completion window, resulted in unenrollment. Completed responses were tracked by MTurk worker ID and IP address to certify that multiple surveys were not completed by the same person. IP addresses were examined via proxycheck to detect proxy or VPN addresses. As no personally identifiable information was collected (except IP addresses, which were deleted following VPN checks), this study was classified as exempt by the National Institutes of Health Intramural Research Program IRB.

Of the 5112 MTurk workers eligible to participate, 3261 (63.8%) began the full survey and 2864 (56.0%) completed it; 249 responses were subsequently removed due to: duplicate IP addresses, indeterminate IP address, IP addresses outside the US, discrepant screener and survey responses for inclusion criteria, and improbably short survey completion times. The final evaluable sample consisted of 2615 complete responses: 1630 (62.3%) reporting past-6-month alcohol-only use and 985 (37.7%) reporting past-6-month use of at least one opioid or stimulant. For this analysis, we examined only responses from participants who reported past-*year* kratom use (N = 174; 6.7% of the 2615 evaluable surveys).

Because data collection occurred during the COVID-19 pandemic, we included some survey items (described below) to assess changes related to the pandemic and its consequences. Those items made the current analysis possible.

### Measures

#### Demographic characteristics

Demographic characteristics were measured using a locally developed set of questions on age, sex/gender, race/ethnicity, education, past-year employment, past-year annual household income, and past-year urban/suburban (vs rural) residence. The latter was measured by converting zip codes into counties and categorizing using the Department of Agriculture’s 2013 Rural-Urban Continuum, where participants who resided in or adjacent to urban/metropolitan areas of ⩾250 000 people were coded “urban/suburban” (vs rural).^68^

#### Psychological-health indicators

Psychological-health indicators included measures of past-month anxiety, depression, and perceived stress. *Past-month anxiety* was measured using the Generalized Anxiety Disorder Scale (GAD-7),^69^ a 7-item assessment of GAD symptoms based on DSM-IV diagnostic criteria. Ratings are made on 4-point Likert scales, with higher values representing greater severity (range: 0-21). *Past-month depression* was measured using the Center for Epidemiologic Studies Short Depression Scale (CES-D-R-10), a 10-item version of the 20-item CES-D,^70-72^ which was modified to assess past-month, rather than past-week, depression in terms of DSM-5 criteria. Items are measured using 4-point Likert scales (range: 0-30), with higher values representing greater depressive symptomatology. Perceived stress was measured using the Perceived Stress Scale (PSS),^73^ a 14-item measure of self-rated past-month stress and coping ability rated on a 4-point Likert scale, with higher values representing greater stress (range: 0-56).

#### SUD symptom severity

SUD symptom severity was assessed by having respondents complete a DSM-5 checklist for the substance that they identified as their biggest problem during the past year (alcohol was one option). Those who responded they had no substance-related problems were asked to complete the DSM-5 checklist for the substance they used the most frequently in the past year. A total score was summed and recoded: “mild” (2-3), “moderate” (4-5), or “severe” (⩾6). For most respondents, DSM SUD items were administered either for alcohol (n = 46), kratom (n = 27: 14 “biggest problem drug” and 13 “most frequently used”), cannabis (n = 26), tobacco (n = 24), or non-kratom opioids (n = 16). Analyses here will not focus on kratom-specific DSM SUD responses, as these were assessed in detail elsewhere.^74^

#### COVID-19-related changes in drug-use amounts

COVID-19-related changes in drug-use amounts were assessed by asking all participants who endorsed past-year and/or past-month of a given drug: “Has your use of [drug] increased or decreased since the COVID-19 pandemic began?” to which people could respond “Increased,” “Decreased,” “No change,” or “I don’t know.”

#### COVID-19-related changes in drug use for the better or worse

COVID-19-related changes in drug use for the better or worse were assessed by asking all participants who endorsed past-year and/or past-month of a given drug: “Has your use of [drug] changed for the worse as a result of the COVID-19 pandemic?” to which they could respond on a 5-point Likert scale (“Not at all” = 0 to “Extremely” = 4); “Has your use of [drug] changed for the better as a result of the COVID-19 pandemic?” to which they could respond on a 5-point Likert scale (“Not at all” = 0 to “Extremely” = 4). To make data analysis and presentation more manageable, we collapsed these response categories into “not at all” versus all other categories. The nuance that may have been lost by that decision was, we believe, mitigated by our analysis of open-ended responses.

### Statistical analysis

All statistical analyses were conducted in R (v 4.1.1) using the following libraries: “lme4,”^75^ “lmtest,”^76^ “arsenal,”^77^ and “sjPlot.”^78^ To examine whether reports of pandemic-related changes “for the better” and “for the worse” differed by substance and whether people increased or decreased use of that substance as a result of the pandemic, we used 2 generalized linear mixed effects regression models (GLMER). GLMERs differ from ordinary least squares (OLS) regression models in their ability to model both fixed and random effects and in their ability to model a wide range of response variable distributions.^75,79^ Here, we modeled dichotomous response variables using a binomial distribution and logit link function; the “lme4” R package employs adaptive Gauss–Hermite quadrature for maximum likelihood approximation.^75^ These binary response variables were *changes for worse* relative to *no change* and *changes for the better* relative to *no change*. The primary explanatory variables of interest were contrast comparisons between kratom and the other most prevalent substances used in our sample (ie, alcohol, cannabis, tobacco, nonmedical opioids, and nonmedical stimulants) and their interaction with pandemic-related changes in the amount of substance used (ie, increases vs no change, decreases vs no change). Because these substance use reports were nested within participants and within differing time periods (ie, past-month and past-year), participants and time were first evaluated as random effects using likelihood ratio tests. We controlled for participant demographic and psychosocial characteristics (ie, factors listed in Table 1), as these were initially entered as fixed effect covariates, and which were retained if chi-square model comparison tests indicated that they significantly improved model fit. Models were tested for overdispersion by comparing Pearson residuals extracted from each model to a chi-square distribution with the same degrees of freedom, but none required correction to meet distribution assumptions. Akaike (AIC) and Bayesian Information Criteria (BIC) were used to evaluate goodness of model fit, and Intraclass Correlation Coefficients (ICCs) were used to estimate the proportion of variance accounted for by model random effects.^80^

**Table 1.** Sample demographic characteristics, psychological health indicators, and past-year substance use (N = 174).

	N	
Age		34.6 (SD 8.7)
Sex/Gender		
Male	87	50%
Female	81	47%
Non-Binary	6	3%
Race/Ethnicity		
White	124	71%
Black/African American	10	6%
Hispanic	17	10%
Asian	13	7%
Other	10	6%
Education		
HS Graduate	115	66%
College Graduate	57	33%
Past-year employment		
Full-time	84	48%
Part-time	34	20%
Unemployed	49	28%
Student	7	4%
Psychological health indicators		
Past-month anxiety, GAD-7 Total Score (0-21)		9.7 (SD 7.2)
Past-month depression, CES-D-R-10 (0-30)		14.2 (SD 6.9)
Past month perceived stress, PSS Total Score (0-56)		32.5 (SD 8.2)
Substance use disorder severity		
None	30	17%
Mild	27	16%
Moderate	36	21%
Severe	81	47%

GAD-7 scores greater than 5, 10, and 15 are indicative of mild, moderate, and severe anxiety symptoms, respectively. CES-D-R-10 scores >16 indicate clinical depressive symptoms. PSS scores greater than 27 denote high perceived stress.

### Text analysis of open-ended responses

Respondents who indicated any change (ie, any response other than “Not at all” were then presented with an optional open-ended prompt: “If you’d like to describe how your use of [drug] has changed for the better [or worse, depending on their response] as a result of the COVID-19 pandemic, please do so below.” For the open-ended responses on changes in kratom use, we identified 9 expected themes a priori based on prior survey findings,^35,36^ findings from mixed-methods studies on kratom use during the COVID-19 pandemic,^81^ and our own recent analyses of Reddit posts pertaining to kratom^52^ and tianeptine (which we found was often used contemporaneously with kratom).^53^ We expected that changes in use “for the better” would mostly correspond to decreases in amount used, and that changes “for the worse” would mostly correspond to increases in amount used. Two independent raters (J.R. and D.S.) read all open responses. After conferencing, a codebook containing 11 codes was finalized. This includes 2 codes indicating whether the valence of the open response was generally “negative” or “positive” in its description of COVID-19-related changes in use. J.R. and D.S. independently coded all text using MAXQDA 2021 (VERBI Software, Berlin).

The open-ended responses often contained multiple types of information. Raters were instructed to apply codes to any relevant text. Thus, multiple codes could be applied to the same text segment. As total percent agreement was high, raters did not conference and subsequently recode text to achieve a higher agreement rate. Additionally, we did not approach this project as an in-depth qualitative analysis and were not seeking to perform iterative coding and sampling for generating theory. Rather, we wanted to characterize and contextualize self-reported increases or decreases in kratom use within the context of COVID-19.

## Results

### Demographic characteristics, psychological health indicators, and past-year substance use

Table 1 shows demographic characteristics, psychological-health indicators, and past-year substance use for respondents who reported past-year kratom use (N = 174). The sample was on average 34.6 (SD 8.7, range 19-62) years old, half male (50%), predominantly White (71%), high-school educated (64%) or college educated (34%), and employed either full-time (48%) or part-time (20%). Approximately 57% reported earning <$35 000 in household income during the past year. Most (78%) resided in urban/suburban settings during the past year.

The past-month mean anxiety symptom score on the GAD-7 was 9.7 (SD 7.2) out of 21, indicating moderate anxiety,^69^ while the past-month mean depressive symptom score on the CES-D-R-10 was 14.2 (SD 6.9) out of 30, indicating moderate to severe symptoms of clinical depression.^70,82^ The past-month mean perceived stress score on the PSS was 32.5 (SD 8.2) out of 56, indicating high (rather than low or moderate) stress.

Most respondents (82.8%) met criteria for at least one past-year SUD; for 46.6%, the symptom count was in the “severe” range, and for 20.7% it was in the “moderate” range. Other than kratom, the most commonly used substances were alcohol (n = 156, 89.7%), nonmedical cannabis (n = 128, 73.6%), tobacco (n = 115, 66.1%), nonmedical/diverted opioids (n = 69, 40.0%), and nonmedical/diverted stimulants (n = 52, 29.9%).

### COVID-19-related changes in amounts of past-year and past-month drug use

Table 2 shows pandemic-related changes in use of kratom, alcohol, cannabis, tobacco, non-medical opioids, and non-medical stimulants. Open-ended items were optional and not all 174 respondents completed them. Thus, sample sizes within each question ranged from 23 for past-month nonmedical stimulants to 174 for past-year kratom. Overall, participants indicated that their amounts of substance use did change as a result of COVID-19. However, the direction of change varied by drug and time period: for most respondents, changes had occurred during the past year, but not as frequently in the past month.

**Table 2.** Counts and percentages of responses to Covid-19-related kratom, tobacco, alcohol, cannabis, opioid, and stimulant use changes and perceptions of change for “the better” or for “the worse” for past-year and past- month time periods (N = 174).

Use in the past year—n (%)						Use in the past month—n (%)					
Kratom (n = 174)		Decreased	Increased	No Change	I don’t know	Kratom (n = 115)		Decreased	Increased	No Change	I don’t know
	Better	23 (13.2)	14 (8)	14 (8)	0 (0)		Better	10 (8.7)	4 (3.5)	6 (5.2)	0 (0)
	Both	1 (0.6)	8 (4.6)	2 (1.1)	0 (0)		Both	0 (0)	4 (3.5)	0 (0)	0 (0)
	Neither	16 (9.2)	14 (8)	54 (31)	1 (0.6)		Neither	2 (1.7)	9 (7.8)	67 (58.3)	0 (0)
	Worse	2 (1.1)	22 (12.6)	3 (1.7)	0 (0)		Worse	0 (0)	13 (11.3)	0 (0)	0 (0)
Tobacco (n = 115)		Decreased	Increased	No Change	I don’t know	Tobacco (n = 80)		Decreased	Increased	No Change	I don’t know
	Better	25 (21.7)	1 (0.9)	4 (3.5)	0 (0)		Better	10 (12.5)	0 (0)	0 (0)	0 (0)
	Both	0 (0)	3 (2.6)	0 (0)	0 (0)		Both	0 (0)	3 (3.8)	0 (0)	1 (1.3)
	Neither	5 (4.3)	2 (1.7)	40 (34.8)	1 (0.9)		Neither	4 (5)	1 (1.3)	35 (43.8)	1 (1.3)
	Worse	0 (0)	33 (28.7)	1 (0.9)	0 (0)		Worse	0 (0)	25 (31.3)	0 (0)	0 (0)
Alcohol (n = 156)		Decreased	Increased	No Change	I don’t know	Alcohol (n = 130)		Decreased	Increased	No Change	I don’t know
	Better	40 (25.6)	0 (0)	8 (5.1)	1 (0.6)		Better	26 (20)	1 (0.8)	5 (3.8)	0 (0)
	Both	0 (0)	3 (1.9)	0 (0)	0 (0)		Both	1 (0.8)	2 (1.5)	0 (0)	0 (0)
	Neither	6 (3.8)	7 (4.5)	47 (30.1)	0 (0)		Neither	5 (3.8)	2 (1.5)	63 (48.5)	0 (0)
	Worse	2 (1.3)	40 (25.6)	2 (1.3)	0 (0)		Worse	1 (0.8)	22 (16.9)	2 (1.5)	0 (0)
Cannabis (n = 128)		Decreased	Increased	No Change	I don’t know	Cannabis (n = 105)		Decreased	Increased	No Change	I don’t know
	Better	10 (7.8)	15 (11.7)	5 (3.9)	0 (0)		Better	9 (8.6)	12 (11.4)	5 (4.8)	0 (0)
	Both	1 (0.8)	9 (7)	1 (0.8)	0 (0)		Both	0 (0)	3 (2.9)	0 (0)	0 (0)
	Neither	12 (9.4)	10 (7.8)	41 (32)	1 (0.8)		Neither	6 (5.7)	2 (1.9)	43 (41)	1 (1)
	Worse	3 (2.3)	18 (14.1)	2 (1.6)	0 (0)		Worse	1 (1)	19 (18.1)	4 (3.8)	0 (0)
Stimulants (n = 52)		Decreased	Increased	No Change	I don’t know	Stimulants (n = 23)		Decreased	Increased	No Change	I don’t know
	Better	14 (26.9)	2 (3.8)	2 (3.8)	0 (0)		Better	1 (4.3)	0 (0)	1 (4.3)	0 (0)
	Both	0 (0)	2 (3.8)	0 (0)	0 (0)		Both	0 (0)	2 (8.7)	0 (0)	0 (0)
	Neither	6 (11.5)	2 (3.8)	15 (28.8)	2 (3.8)		Neither	3 (13)	2 (8.7)	10 (43.5)	0 (0)
	Worse	0 (0)	6 (11.5)	1 (1.9)	0 (0)		Worse	1 (4.3)	3 (13)	0 (0)	0 (0)
Opioids (n = 69)		Decreased	Increased	No Change	I don’t know	Opioids (n = 42)		Decreased	Increased	No Change	I don’t know
	Better	15 (21.7)	3 (4.3)	3 (4.3)	0 (0)		Better	6 (14.3)	2 (4.8)	2 (4.8)	0 (0)
	Both	0 (0)	2 (2.9)	0 (0)	0 (0)		Both	1 (2.4)	1 (2.4)	0 (0)	0 (0)
	Neither	5 (7.2)	5 (7.2)	18 (26.1)	1 (1.4)		Neither	3 (7.1)	1 (2.4)	18 (42.9)	1 (2.4)
	Worse	4 (5.8)	11 (15.9)	2 (2.9)	0 (0)		Worse	0 (0)	5 (11.9)	2 (4.8)	0 (0)

Percentages use the full denominator for each set of questions.

Proportions of participants reporting changes in past-year drug use were well distributed across increases, decreases, and no change. For kratom 33% (n = 58) reported a COVID-related increase and 24% (n = 42) reported a COVID-related decrease. For tobacco, 34% (n = 39) reported a COVID-related increase and 26% (n = 30) reported a COVID-related decrease. For alcohol, 32% (n = 49) reported a COVID-related increase and 31% (n = 48) reported a Covid-related decrease. For cannabis, 41% (n = 52) reported a COVID-related increase and 20% (n = 26) reported a COVID-related decrease. For nonmedical stimulants, 23% (n = 12) reported a COVID-related increase and 38% (n = 20) reported a COVID-related decrease. Finally, for nonmedical opioids, 30% (n = 21) reported a COVID-related increase and 35% (n = 24) reported a COVID-related decrease.

### GLMER models of changes in use: For the better or worse

**Figure 2.**
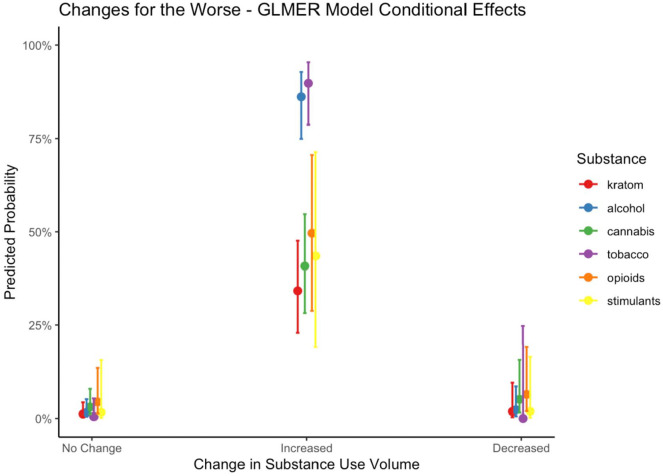
Point estimates and 95% confidence interval for generalized linear mixed effects regression model-fitted probability that substance use had changed for the worse as a result of the COVID-19 pandemic. Marginal effects of substance (eg, kratom, alcohol, etc.) are conditional upon changes in substance use volume.

**Figure 3.**
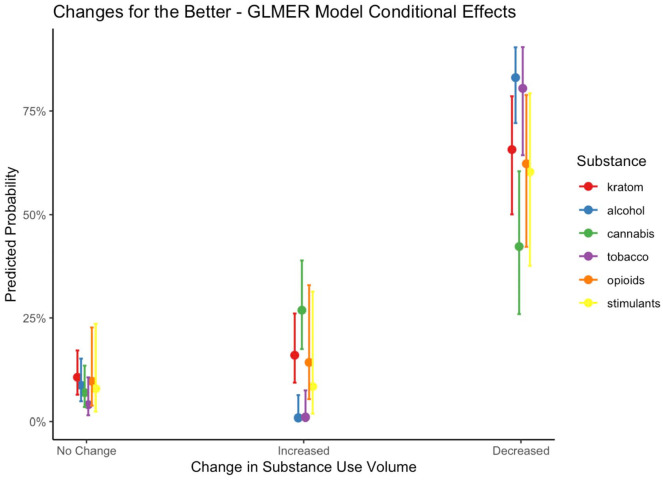
Point estimates and 95% confidence interval for generalized linear mixed effects regression model-fitted probability that substance use had changed for the better as a result of the COVID-19 pandemic. Marginal effects of substance (eg, kratom, alcohol, etc.) are conditional upon changes in substance use volume.

GLMER models were used to examine whether reports of changes for the better and changes for the worse differed by substance and whether the amount used changed as a result of the COVID-19 pandemic. Model fit indices, fixed effect estimates (reported as odds ratios), and random effect estimates (reported as ICCs) can be referenced in Table 3. Conditional model effects resulting from change in amount × substance interactions are represented in Figures 2 and 3. Modeling participants as a random factor accounted for a significant portion of variance in both “better” (χ^2^(1) = 31.2, *P* < .001) and “worse” (χ^2^(1) = 26.2, *P* < .001) initial model comparisons, but modeling time reference frame as a random effect did not improve model fit, and it was not retained in subsequent models.

**Table 3.** Model fit indices, fixed effects estimates, and random effects estimates for generalized linear mixed effect regression (GLMER) models of substance use “changes for the worse” and “changes for the better” as a function of substance type and changes in the amount of substance used, holding constant participants’ demographic and psychosocial characteristics.

“Changes for the Worse” model—main effects					“Changes for the Better” model—main effects				
**Type**	GLMER	**AIC**		716.02	**Type**	GLMER	**AIC**		1003.16
**Family**	Binomial	**BIC**		766.83	**Family**	Binomial	**BIC**		1053.96
**Link**	Logit	**Ps-R² (fixed)**		0.52	**Link**	Logit	**Ps-R² (fixed)**		0.31
		**Ps-R² (total)**		0.65			**Ps-R² (total)**		0.45
Fixed effects	OR	95% CI	z	*P*	Fixed effects	OR	95% CI	z	*P*
**DSM-5 SUD Symptoms**	1.16	[1.07, 1.27]	3.44	<**.001**	**Age**	**0.97**	**[0.94, 0.99]**	−2.27	**.02**
*Substance Amount*					*Substance Amount*				
**No Change—Increased**	83.90	[42.35, 166.21]	12.70	<**.001**	No Change - Increased	1.45	[0.93, 2.27]	1.64	.10
No Change—Decreased	1.33	[0.61, 2.9]	0.71	.47	**No Change - Decreased**	**26.08**	**[16.29, 41.74]**	**13.59**	<**.001**
*Substance*					*Substance*				
**Kratom—Alcohol**	5.02	[2.57, 9.81]	4.71	<**.001**	Kratom - Alcohol	0.8	[0.49, 1.29]	−0.91	.36
Kratom—Cannabis	1.77	[0.9, 3.48]	1.66	.10	Kratom - Cannabis	0.89	[0.54, 1.46]	−0.46	.65
**Kratom—Tobacco**	4.72	[2.32, 9.59]	4.29	<**.001**	**Kratom - Tobacco**	**0.53**	**[0.3, 0.94]**	−2.18	**.03**
**Kratom—Opioids**	3.42	[1.46, 8.02]	2.83	<**.001**	Kratom - Opioids	0.76	[0.39, 1.46]	−0.82	.41
Kratom—Stimulants	1.56	[0.52, 4.62]	0.80	.43	Kratom - Stimulants	0.59	[0.27, 1.27]	−1.35	.18
Random effects					Random effects				
Group	ICC	SD			Group	ICC	SD		
Subject (N = 174)	0.27	1.10			Subject (N = 174)	0.20	0.90		

Statistically significant fixed effects are indicated in bold.

AIC, Akaike information criterion; BIC, Bayesian information criterion; ICC, intraclass correlation coefficient; OR, odds ratio; Ps-R^2^, Pseudo R-squared.

#### Changes for the worse

Modeling participants as random subject effects accounted for approximately 27% of the variance in reports of changes for the worse (ICC = 0.27). Of the demographic and psychosocial characteristics included as covariates in the model, only DSM-5 SUD symptoms were associated with changes for the worse. Each additional SUD symptom reported was associated with a 16% increase in the odds of reporting a change for the worse (OR = 1.16). Controlling for DSM-5 symptom counts and substance used, increases in substance use were associated with 82.9 times the odds of being “for the worse” compared with no change in amount (OR = 83.90), and decreased substance use was not significantly associated with changes for the worse. Controlling for changes in amount used, alcohol (OR = 5.02), tobacco (OR = 4.72), and nonmedical opioid use (OR = 3.42) were all more likely to have changed for the worse, compared with kratom use.

Modeling the interaction between substance contrast comparisons and changes in amount resulted in improved model fit as indicated by likelihood ratio model comparison (χ^2^(10) = 44.8, *P* < .001). Model fitted probability estimates and 95% confidence interval of changes for the worse are displayed in Figure 2. Relative to increases in kratom use, increases in alcohol (OR = 8.79) and tobacco (OR = 40.84) use were significantly more likely to be changes for the worse than increases in kratom use. No significant differences were observed between increases in kratom and increases in nonmedical opioids, nonmedical stimulants, or cannabis. There were no significant decrease × substance interactions.

#### Changes for the better

Modeling participants as random subject effects accounted for approximately 20% of the variance in reports of changes for the better (ICC = 0.20). Of the demographic and psychosocial characteristics included as covariates in the model, only age was significantly associated with changes for the better, such that the odds of changes for the better decreased by 3% for every 1-year increase in age (OR = 0.97). Controlling for age and substance, decreased amount was more likely to be associated with changes for the better (OR = 26.08), and there was no significant main effect of increased amount. Controlling for changes in amount, tobacco use was 47% less likely than kratom use to change for the better (OR = 0.53).

Modeling the interaction between substance contrast comparisons and changes in amount resulted in improved model fit as indicated by likelihood ratio model comparison (χ^2^(10) = 65.4, *P* < .001). Model fitted probability estimates and 95% confidence interval of changes for the better are displayed in Figure 3. Relative to decreases in kratom use, decreases in alcohol (OR = 3.21) and tobacco (OR = 6.18) use were more likely to be changes for the better. Additionally, increased alcohol use was 94% less likely than increased kratom use to be a change for the better (OR = 0.06). Increased cannabis use was more likely than increased kratom use to be a change for the better (OR = 3.09).

### COVID-related changes in kratom use contextualized in open-ended responses

Nearly half of respondents completed the optional open-ended items on COVID-19-related changes in kratom use (49.1%; n = 85). Table 4 shows thematic codes and interrater agreements and disagreements.

**Table 4.** All codes, interrater agreements and disagreements, agreement percent, and total number of codes applied to the optional open-text survey items pertaining to COVID-19-related kratom use changes (n = 85).

Code	Agree	Disagree	Total number of codes applied	Agreement %
Self-treat pain symptoms	16	0	16	100.0
Self-treating mood, anxiety, emotional distress, stress symptoms	28	3	31	90.3
Use for energy or enhancing general wellness	28	6	34	82.4
Kratom as a substitute for other drugs	30	2	32	93.8
Self-treating other drug withdrawal	16	1	17	94.1
Kratom reduces other drug craving	10	1	11	90.9
Boredom	8	0	8	100.0
Difficulty obtaining kratom during Covid-19	16	0	16	100.0
Adverse effects	20	0	20	100.0
Negative	24	6	30	80.0
Positive	46	0	46	100.0
Total	242	19	261	94.7

A total of 261 codes were applied to the 85 open-response texts; 242 (94.7%) were concordant and 19 (5.35%) were discordant, with an average of 4.18 codes assigned per response entry. Agreement was 100% for the 2 additional codes applied to classify text as reflecting increases or decreases (and thus corresponding to the stem question). Agreement was also high for the codes reflecting valence (“negative” or “positive”), with slightly more responses coded as positive (N = 46/85) than negative (N = 30/85).

Many of the motivations that respondents cited for their increases or decreases in kratom use during COVID-19 correspond to motivations for kratom use that have been discussed in prior literature, including: use as a replacement for other drugs (N = 30/85); use as a self-treatment for withdrawal symptoms from other drugs (N = 16/85); use to reduce craving for other drugs (10/85), including opioids; and use as a self-treatment for physical pain (N = 16/85). Motivations also included self-treating problems with anxiety, stress, or low mood (N = 28/85), as well as increasing energy and enhancing general wellness or sense of well-being (N = 28/85). More specific to the pandemic, and reported by a minority, included increasing kratom use to address feelings of boredom (N = 8/85) or decreasing use due to decreases in availability during COVID-19 (N = 16/85). Importantly, adverse kratom effects were also described (N = 20/85).

## Discussion

Our models of substance use “changes for the better” and “changes for the worse” indicate that when controlling for substance type, increases were strongly related to changes for the worse, and decreases were strongly related to changes for the better. However, both models were improved with the inclusion of a change in amount × substance interaction term, indicating that the relationship between change in amount and change for the better/worse is conditional on the substance in question. In particular, the relationship between increased use and change for worse appears to be driven primarily by alcohol and tobacco, as increased use of these substances was much more likely to be associated with changes for the worse, relative to kratom use. As shown in Figure 2, increases in kratom and cannabis use displayed the lowest model-fitted probability of changes for the worse, these being 34% and 41%, respectively. A similar pattern was observed in changes for the better, as decreases in alcohol and tobacco use were far more likely than kratom to be associated with changes for the better. Shown in Figure 3, decreases in kratom were less likely than alcohol and tobacco but equally likely as nonmedical opioids and stimulants to be changes for the better. Cannabis use was the only substance to display a probability lower than 50% of being a decrease for the better, and of the increases, cannabis use displayed the highest probability of being for the better.

The dissociation between amount of use and unhealthiness of use, at least for kratom, cannabis, and nonmedical stimulants, is consistent with a longstanding recognition, in the assessment of SUDs, that there is not necessarily an inevitable correspondence between *more* (or *more often*) and *worse*. DSM criteria are predicated on that distinction: they rely on amount or frequency of use only inasmuch as those measures are consequential in context (such as using more drug than intended, or spending time on drug use to the exclusion of other valued activities). The current findings underscore the pitfalls of inferring increases in problematic use from data that assess only amount or frequency of use: the 2 are at least partly orthogonal. This orthogonality was less present in our data for alcohol and tobacco than for kratom. Respondents never judged that their increases in drinking represented a change entirely for the better (though 3 respondents judged that their increases in drinking were part of a mixed pattern of changes for better and worse), and only one participant indicated a past-year tobacco increase for the better. This might partly reflect absorbed cultural messages about alcohol, but is also consistent with recent findings that negative consequences of drinking, on any given occasion, do appear to vary monotonically with the number of drinks consumed, at least for young adults.^83^

The complexity of changes in kratom use, especially during COVID-19, is underscored by the open-ended responses we obtained from our sample. Although a majority reported changes in kratom use because of the pandemic, including difficulty obtaining kratom (due to economic conditions or supply availability) and due to pandemic-related psychological stressors or boredom, some people described kratom use that was not clearly tied to the pandemic, such as using to mitigate opioid or other drug withdrawal, or decreasing use due to adverse effects. It thus remains unclear the degree to which people increased or decreased kratom use directly due to pandemic effects as opposed to physical and psychological health conditions and circumstances that happened to coincide with a pandemic. Our findings are in keeping with survey and social-media findings indicating that COVID-19 did not profoundly disrupt (or profoundly increase) kratom use for most people who had been using it, but that a mixed pattern of increases and decreases did occur, for a panoply of reasons.^81^

Despite the fact that the US kratom market was not greatly impacted by the pandemic in 2020, the absence of a disruption was far from certain at first. As we were able to assess past-month psychological symptoms among this sample, one clearer set of findings includes the fact that most people in this sample of past-year kratom-using adults not only qualified for an SUD, but also scored high on measures of anxiety, stress, and depression. Pandemic or not, they were experiencing many symptoms characteristic of diminished well-being (ie, elevated anxiety, depressive symptoms, and perceived stress) that we increasingly recognize as a broad motivation for kratom use.^4,6-8^ It is likely that COVID-19 added yet another layer of complexity in the lives of people who were already experiencing a fair degree of it, and that uncertainty about the continued availability of kratom was an unwanted complexity. Circumstantial complexity is evidenced in participants’ open responses here and elsewhere,^52^ and it suggests that in addition to more rigorous methods of investigation needed in the study of kratom, including longitudinal study and controlled laboratory experiments, narrative qualitative methods are warranted.

### Limitations

Like any online convenience sample, ours was not representative of everyone in the population of interest (which, for us, was everyone in the US who used or specifically stopped using kratom during the COVID-19 pandemic of 2020). Our sample was similar in several respects to prior kratom-using survey samples in terms of age, race/ethnicity, and distribution of sex/gender. However, our sample had slightly lower past-year annual income than has been found in large online kratom surveys.^35,36^

The limitations associated with a non-kratom-specific study are accompanied by a strength. We were able to obtain what we believe may be a slightly more diverse or representative sample of adults with kratom-use histories and with potentially less self-selection bias. For instance, some people who reported decreased used also reported having quit kratom within the past year, meaning that not all people who reported past-year kratom use were current, users, which distinguishes them from prior online survey samples of regular users who have reported largely positive use experiences with few adverse effects or indicators of kratom withdrawal or addiction.^35^ In some ways, such as motivations for use described in open-text responses, our sample is similar to those from large surveys but, in others, more similar to samples from smaller in-person surveys and social-media analyses: those samples have higher degrees of polysubstance use and of kratom tolerance, withdrawal, and perceived addiction.^35,36,38,51-54,84^ This may be particularly true in that we found high rates of SUD for any drug with just under half meeting DSM-5 criteria for severe SUD. Because we assessed SUD criteria only for some drugs (which did not include kratom for most respondents), we cannot directly compare our findings to those of studies that attempted to operationalize kratom-use disorder.^35,55,85-87^

## Conclusion

Many participants did change their substance use as a result of COVID-19; overall, increases in substance use were most likely to be changes for the worse, and decreases in substance use were most likely to be changes for the better. However, there was no propensity for either increases or decreases in kratom use, nor was there a resounding narrative indicating that most people believed kratom use increases were “for the better” rather than “for the worse.” Increases in kratom and cannabis use were far less likely than alcohol or tobacco to be changes for the worse, and decreases in kratom and cannabis use were far less likely than alcohol and tobacco to be “for the better.” These observed differences may speak to the fact that people not only maintain differing relationships with psychoactive drugs, but that distinct types of relationships can be observed among commonly used drugs.

The pandemic continues to change, as does the kratom market and the array of available kratom products. The durability of these findings lies partly in the support they provide for an understanding that kratom is like other psychoactive substances, in that most people can and do change their use based on the balance of positive and adverse effects as conditions and consequences change.
